# Platelet-rich fibrin for wound healing of palatal donor sites of free gingival grafts: Systematic review and meta-analysis

**DOI:** 10.4317/jced.57451

**Published:** 2021-02-01

**Authors:** David-Jonathan-Rodrigues Gusman, Henrique-Rinaldi Matheus, Breno-Edson-Sendão Alves, Amanda-Munarolo-Piacenza de Oliveira, Amanda-Cristine-dos Santos Britto, Vivian-Cristina-Noronha Novaes, Maria-José-Hitomi Nagata, Victor-Eduardo-de Souza Batista, Juliano-Milanezi de Almeida

**Affiliations:** 1 Department of Diagnostic and Surgery – Periodontics Division. São Paulo State University (UNESP), School of Dentistry, Araçatuba; 2 Department of periodontics, University of Western Sao Paulo (UNOESTE), Presidente Prudente, Sao Paulo, Brazil; 3 Department of periodontics, Maringa University Center (UNINGA), Maringa, Parana, Brazil; 4 Department of periodontics, University Center of Santa Fe do Sul (UNIFUNEC), Santa Fe do Sul, Sao Paulo, Brazil

## Abstract

**Background:**

Platelet-rich fibrin (PRF) has been referred to as a second-generation platelet concentrate, associated with improvements on the healing of palatal wounds followed by FGG harvesting. The aim of this systematic review and meta-analysis was to assess the complete wound epithelialization and postoperative pain when PRF was used in palatal wounds following free gingival graft (FGG) harvesting.

**Material and Methods:**

PubMed (Medline), EMBASE and Scopus were searched by two independent individuals up to and including March 2020 in order to identify controlled and randomized controlled clinical trials on the use of PRF at palatal donor sites of FGG. The outcomes assessed were epithelialization and postoperative pain. The risk of bias of the included studies was evaluated using Cochrane Collaboration’s domain-based two-part tool. Random effects meta-analyses were conducted with 95% confidence intervals.

**Results:**

The search strategy identified 555 potentially eligible articles, of which 6 randomized controlled clinical trials were included. In the qualitative analysis, most studies (83.3%) reported lower postoperative pain in treatment groups, while all studies accessing epithelialization demonstrated earlier complete wound closure in groups treated with PRF. The discomfort and complete re-epithelialization were more favorable in groups PRF when compared to control groups (*P*<0.00001).

**Conclusions:**

Within the limits of the present study, it can be concluded that the use of PRF for wound healing of palatal donor sites of FGG may decrease postoperative pain and induce earlier complete wound epithelialization.

** Key words:**Wound healing, oral surgery procedures, pain, postoperative.

## Introduction

The percentage of individuals affected by gingival recessions varies depending on populations, averaging from 30% to 100% (1). In addition, the prevalence and severity of this condition seems to increase with age (1).

Gingival recessions predispose to reduction in the width of keratinized gingiva, aesthetic deficiency and dentin hypersensitivity, leading to pain during patients’ self-care (1). Some therapies are proposed in order to reduce recessions’ negative impact. Free gingival grafts (FGG) and connective tissue grafts (CTG) have been performed to increase the width of keratinized gingiva and for root coverage, aiming reduction or elimination of dentin hypersensitivity and to recover aesthetics (1).

Different autologous sites are eligible to be donors of FGG and CTG, such as edentulous areas, maxillary tuberosity and palatal mucosa (1,2). Among them, the palate is the most usually chosen donor site (3). The surgical intervention for removal of FGG is relatively easy to be performed and enables obtention of substantial amount of tissue (1). However, this procedure is almost always related to, at least, one of the following complications: acute pain, hemorrhage, and bone exposure, which lead to morbidity and discomfort for the patient during the healing process of the donor site (4). It generally takes 2-4 weeks for FGG palatal wounds to heal by secondary intention (5).

Aiming to avoid or to overcome these issues, studies have reported some therapeutic alternatives for enhancement in the repair process and/or to reduce postoperative pain of palatal donor sites of FGG, such as low-level-laser-therapy (LLLT) (6), ozone therapy (6), platelet rich plasma (7), and others.

The platelet rich fibrin (PRF) is referred to as the second generation of platelet concentrates, widely used in modern medicine (8). In dentistry, it has been used to improve the repair process in post-extraction sockets, sinus lifts, periodontal bone defects, and periodontal plastic surgeries (9). The use of PRF in the palate following the removal of FGG was described by randomized controlled clinical trials (10,11) aiming to reduce the postoperative pain and/or to improve healing. However, to the best of our knowledge, no systematic review and meta-analysis was performed on this topic.

Therefore, in order to confirm the hypothesis that PRF could improve both parameters (i.e. healing and pain), the aim of this systematic review and meta-analysis was to assess the complete wound epithelialization and postoperative pain when PRF was used in palatal wounds following FGG harvesting.

## Material and Methods

-Procedure

This review is registered in the PROSPERO database (CRD42019129790), in compliance with the Preferred Reporting Items for Systematic Reviews and Meta-Analyses (PRISMA) statement guidelines (12).

-Information sources, search, study selection

Two independent reviewers (D.J.R.G., V.C.N.N.) conducted an electronic search on the PubMed/Medline, EMBASE, and Scopus databases for articles published in English language, until 02sd March 2020. The key words used were: “palatal wound healing and free gingival graft; donor site wound healing and free gingival graft; donor site wound healing and connective tissue graft; palatal wound healing and connective tissue graft; palatal wound healing and platelet-rich fibrin”. A further manual search was conducted on the reference lists of relevant journals in the field (Journal of Clinical Periodontology, Journal of Periodontology and Journal of Periodontal Research). The authors also performed a search of non-peer-reviewed literature at http://www.opengrey.eu/. All potential abstracts and complete texts were revised for selection of those that met the criteria detailed below. Disagreements between researchers were settled by consensus. Cohen’s kappa coefficient was used to evaluate the agreement between researchers.

In accordance with the PICO framework (12), it was used the focus question: “Can platelet-rich fibrin to improve epithelialization and to reduce postoperative pain at the donor site of FGG?”

• Population: adult patients that underwent surgical removal of FGG from their palates;

• Intervention: adaptation of platelet-rich fibrin at the donor site of FGG;

• Comparison: with their respective control groups (sterile wet gauze pressure, natural wound closure, use of gelatin sponge, butyl-cyanoacrylate, or wound coverage with dressing materials)

• Outcomes: wound epithelialization (percentage of complete wound epithelialization [through H202 bubbling], or analysis of contour changes rated by scores) and postoperative pain (visual analog scale [VAS]).

-Eligibility criteria 

Controlled clinical trials and randomized controlled clinical trials published in the English language, evaluating wound epithelialization and/or postoperative pain at the donor site of FGG in healthy patients.

Articles that failed to meet the inclusion criteria: studies that did not evaluate wound epithelialization or postoperative pain; grafts collected by a different method than the conventional technique described by Sullivan and Atkins (1968) (2) (rectangular graft removal of palatal donor site [epithelium and connective tissue]).

-Data items and data collection process

One reviewer collected information from the selected articles, including author, year of publication, country, type of study, groups evaluated, analyses and evaluation period, preparation of PRF, prescribed medications, the main outcome, and authors’ conclusion. A second reviewer checked all information collected by the first reviewer.

-Risk of bias in individual studies

The risk of bias of the randomized controlled trials (RCTs) included was assessed using the Cochrane Collaboration Tool for Assessing Risk of Bias in Randomized Trials (13).

-Summary measures, risk of bias among the studies, synthesis of results

Meta-analysis was based on the inverse variance (IV) and Mantel-Haenzel (M-H) methods. The discomfort was continuous outcome and assessed by mean difference (MD) values. The complete re-epithelialization of the palatal wound was dichotomous outcome assessed by odds ratio (OR). A random-effects model was used to assess the significance of the treatment effects (14) with corresponding 95% confidence intervals (CI). A computer software (Reviewer Manager 5; Cochrane Group) was used to perform the meta-analysis and to produce the funnel plots.

An asymmetric funnel plot can suggest publication bias or other biases related to sample size, although the asymmetry can show a true association between trial size and effect size (14). Heterogeneity was evaluated by the Q method (x2) and the I2 value. An I2 of <60% was the cut-off for homogeneity of the data, justifying pooling.

## Results

-Literature research

**Figure 1:**
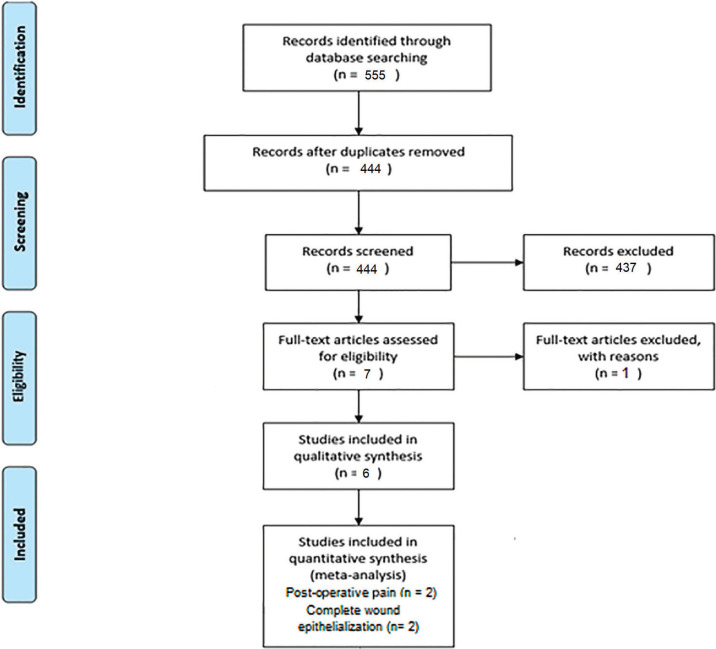
Flow chart of manuscripts screened through the review process.

The electronic search on the databases identified 555 articles (Figure 1 shows details of the research process and studies’ selection). After elimination of duplicates, a total of 444 articles were screened by title and abstract. The articles not fulfilling the PICO framework were considered ineligible. At the end of this procedure, 437 articles were excluded. Thus, seven full-texts were analyzed, and one article was excluded (15) due to the different technique than Sullivan and Atkins (1968) (2) for removal of the graft (single-incision), assigned in the exclusion criteria. Finally, six articles were selected for systematic review, (4,10,11,16,17,18) two articles articles (4,16) for meta-analysis of postoperative pain (VAS), and two articles (10,16) for meta-analysis of complete wound epithelialization (H2O2 bubbling). Cohen’s kappa coefficient indicated 100% of agreement between reviewers.

-Assessment of risk of bias and quality assessment in included studies

**Figure 2:**
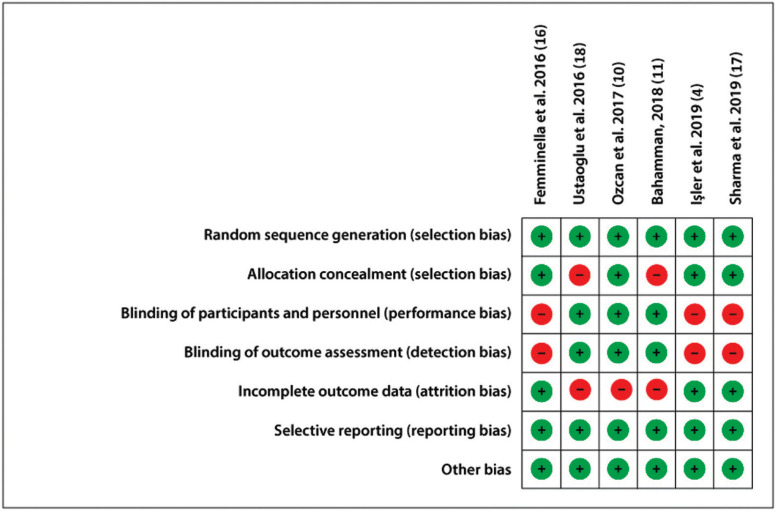
Risk of bias summary.

A summary of the methodological quality assessment of the studies included is described in Figure 2.

-Characteristics of the included studies related to patients

A total of 247 patients were allocated to the control groups and groups that had the palatal wound treated with PRF. Among these patients, 140 were from control groups, consisting of spontaneously secondary healing (4), sterile wet gauze pressure (18), gelatin sponge (17), butyl-cyanoacrylate (10), and bandage with non-eugenol periodontal pack (coe-pak TM) (11) and collagen dressing (CollaCote®) (17). In test groups, 107 patients were treated with PRF bandage (4,16,17), platelet concentrate obtained by centrifugation in titanium tubes (T-PRF) and used as bandage (18), PRF + butyl-cyanoacrylate (10), and PRF bandage + non-eugenol periodontal pack (coe-pak TM) (11).

The mean age of the patients when considering both control and test groups was 34.79 ± 7.87. This data was obtained from 4 studies, since Ustaoglu *et al.* (18) and Sharma *et al.* 2019 (17) did not mention the mean age of their patients. All studies included are randomized controlled clinical trials.

Different medications were prescribed in the experiments. Femminella *et al.* 2016 (16) and Bahamman (11) reported lower use of analgesic in test groups. On the other hand, Ustaoglu *et al.* (18) did not find difference in the amount of analgesics’ intake by the patients between control and test groups. Ozcan *et al.* (10) prescribed no analgesics, and İşler *et al.* (4) presented no data due to the lack of standardization.

Among the studies assessing the VAS, Femminella *et al.* (16), Ozcan *et al.* (10), Bahamman, (11), İşler *et al.* (4), and Sharma *et al.* (17) observed reduction of the postoperative pain in different periods of analysis. Only Ustaoglu *et al.* (18) observed no difference in postoperative pain by using T-PRF.

All studies evaluating the epithelialization of the palatal wound showed that PRF promoted complete healing in shorter time periods when compared with its respective control groups, independent on the method of analysis (peroxide test - H2O2-bubbling [10, 16, 18] or image based scores [by five senior residents in blind periodontics]) (11). The results are described in Table 1,  Table 1 cont.,  Table 1 cont.-1,  Table 1 cont.-2.

**Table 1:**
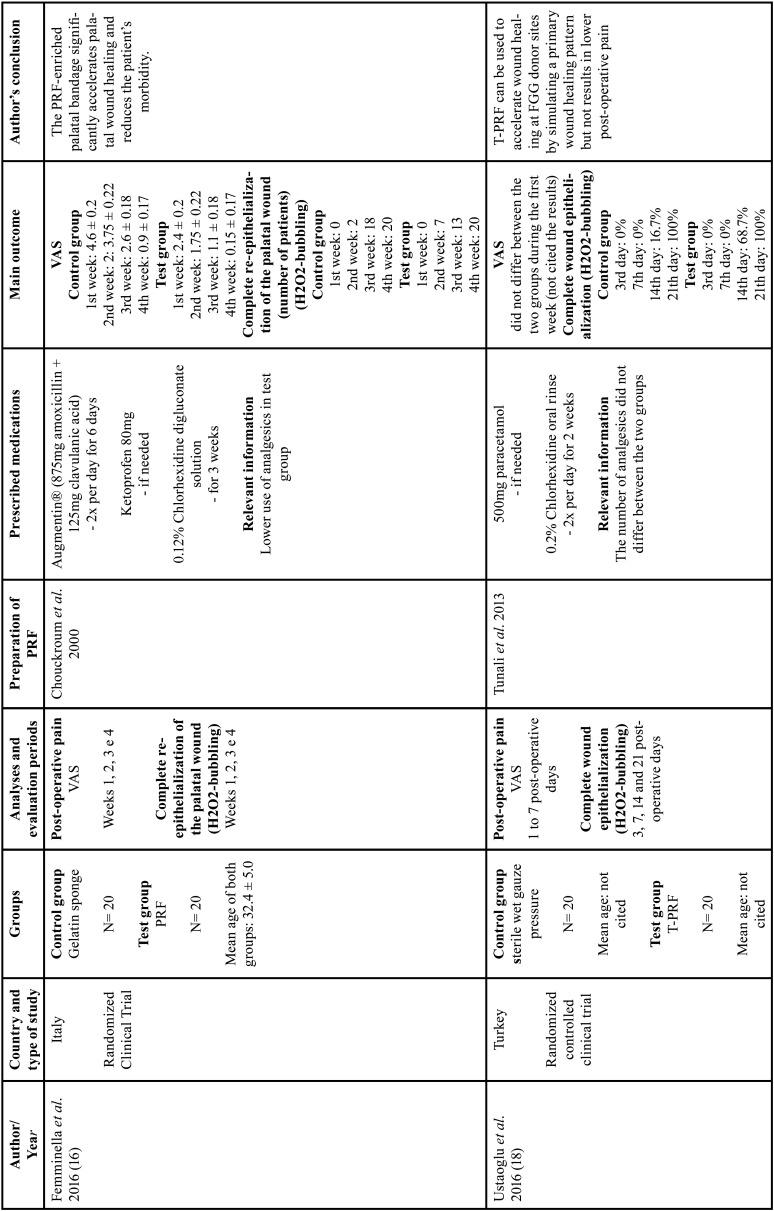
Characteristics of studies included.

**Table 1 cont.**
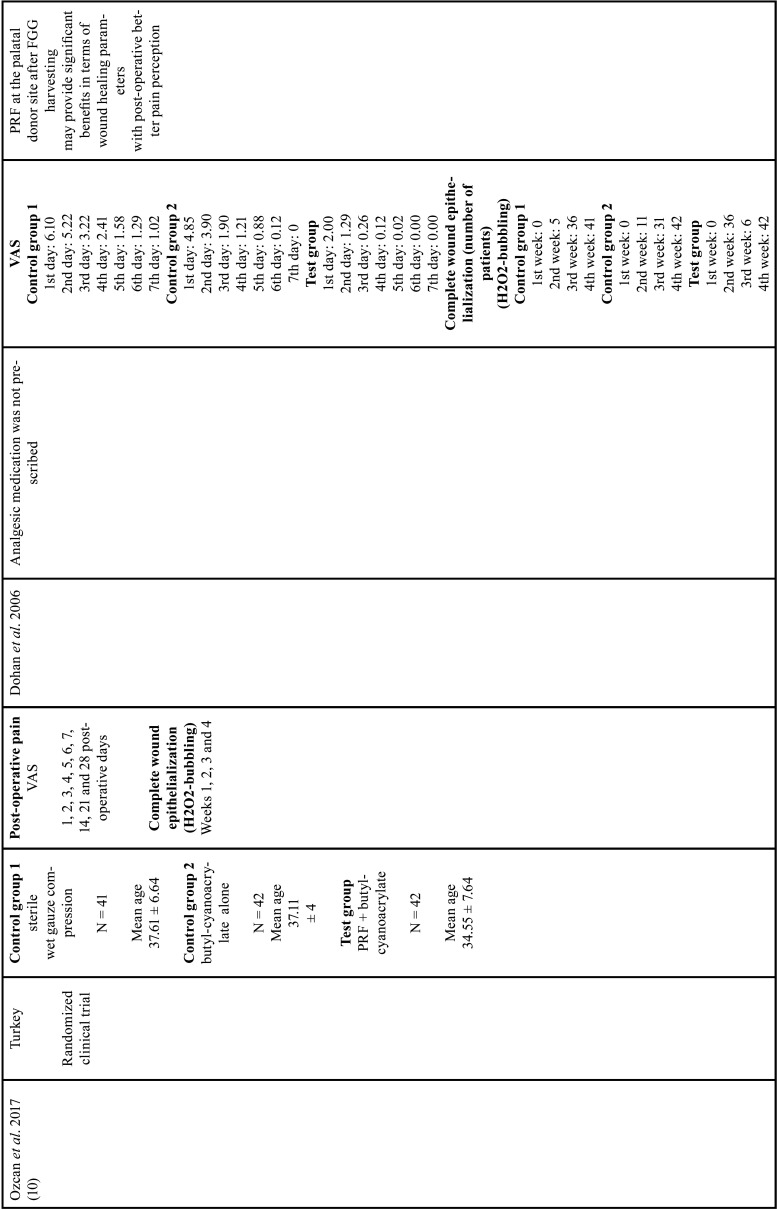
Characteristics of studies included.

**Table 1 cont.-1:**
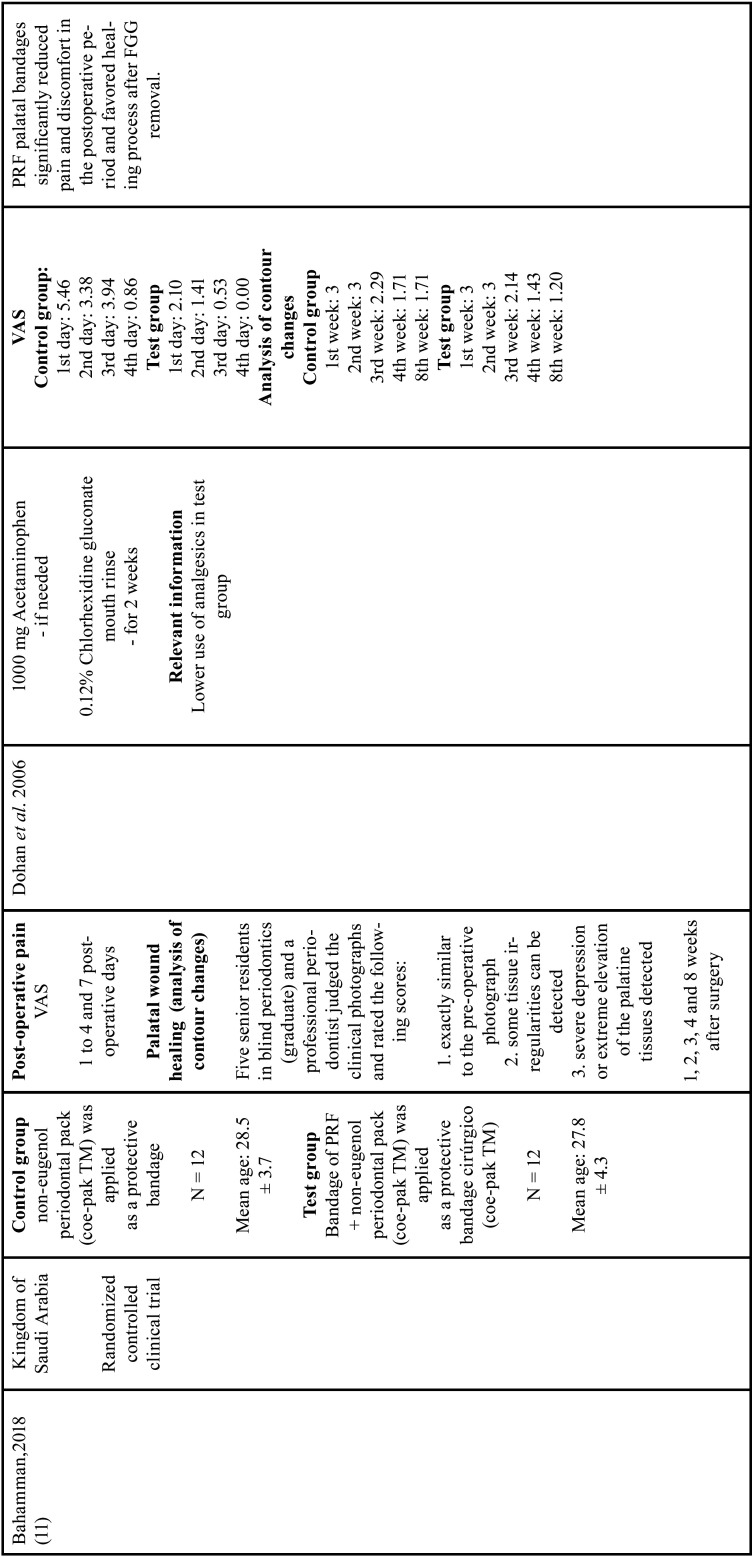
Characteristics of studies included.

**Table 1 cont.-2:**
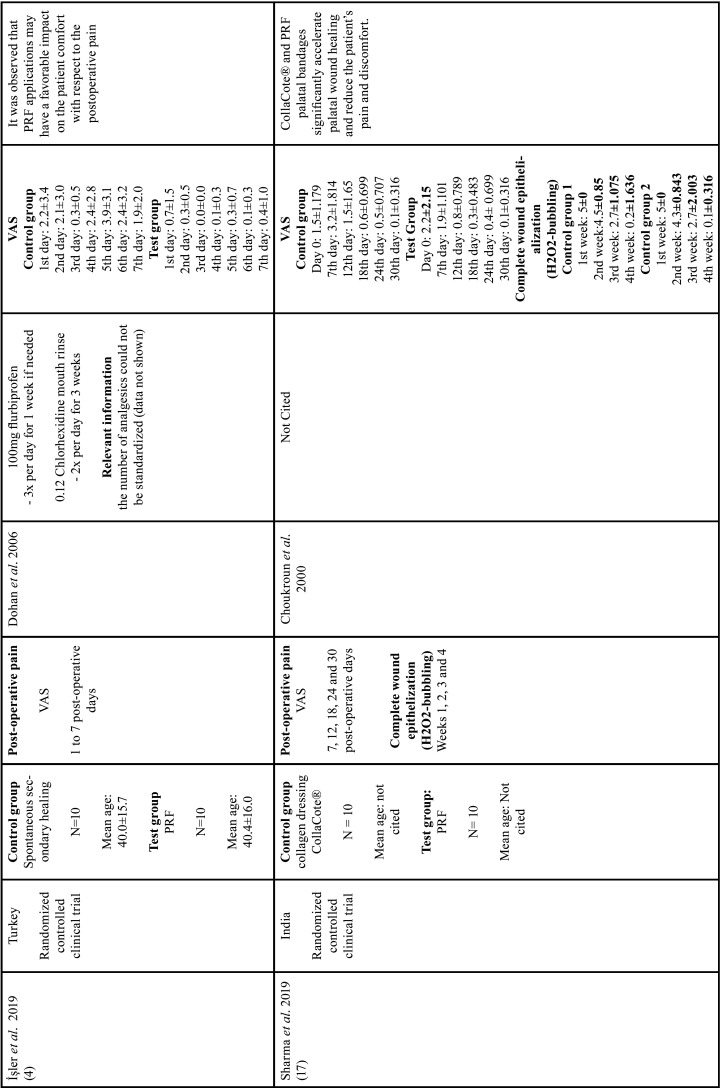
Characteristics of studies included.

-Results of the meta-analysis

**Figure 3:**
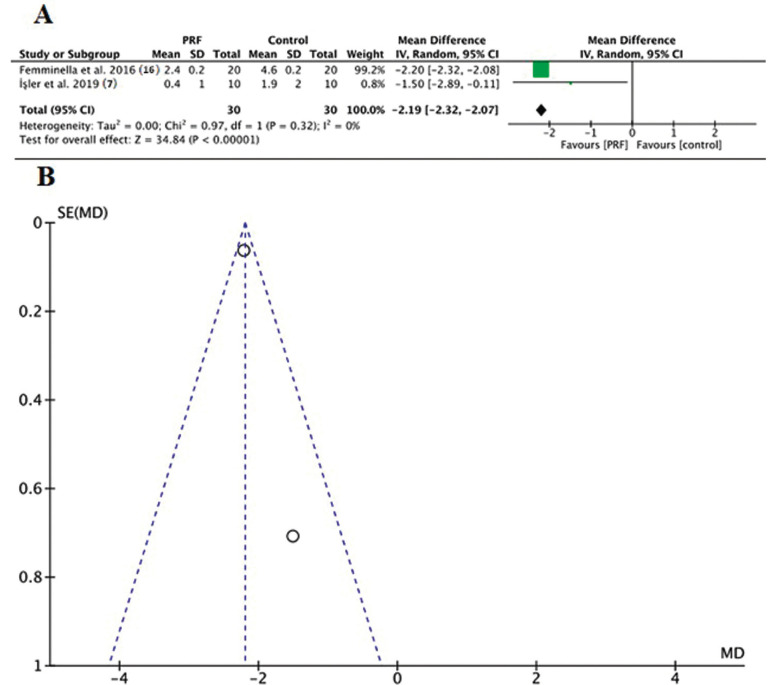
(A) Florest plot. Comparison of studies assessing the discomfort; (B) Funnel plot to evaluate the risk of bias.

**Figure 4:**
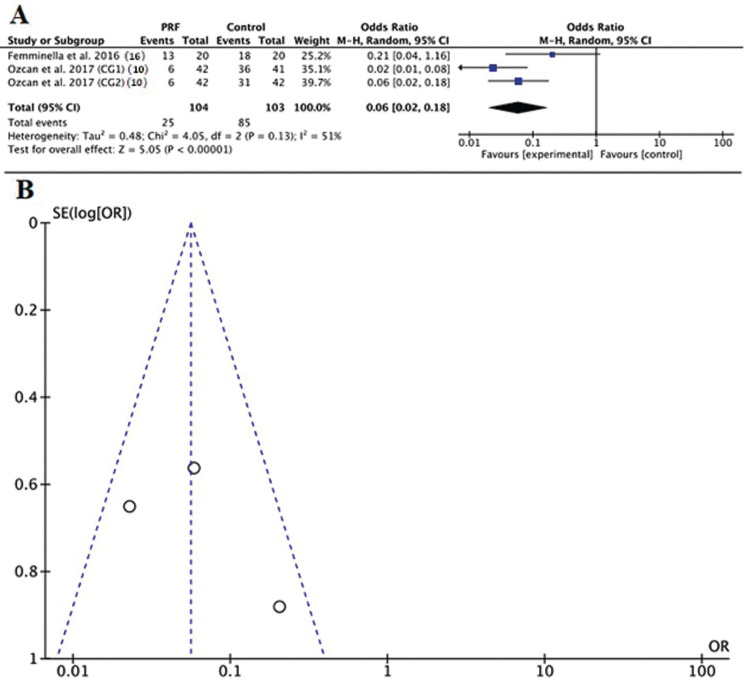
(A) Florest plot. Comparison of studies assessing the complete re-epithelialization of the palatal wound after 14 days; (B) Funnel plot to evaluate the risk of bias.

Two studies used the VAS criteria (4,16) to report the data comparing the interventions. The quantitative analysis indicated difference between the PRF group and control groups (*P*<0.00001) (Fig. 3). Two studies reported the data of complete re-epithelialization of the palatal wound after 14 days (10,16). The quantitative analysis indicated difference between the PRF group and control groups (*P*<0.00001) (Fig. 4). The funnel plots showed evident symmetry among the differences of means in the studies evaluated. The funnel plot showed symmetry in both outcomes (Figs. 3,4).

## Discussion

Normally, complete healing of any wound follows four overlapping phases: hemostasis, inflammation, proliferation, and remodeling (8). These phases are dependent of accurate events involving mediators and signals, guiding specific cells to perform their functions (8). As a cascade, these steps must follow a chronologic order, and, therefore, the first phase plays a determinant role for the completion of wound healing. Platelets are shown as essential cytoplasmatic acellular fragments (19) to regulate the homeostasis phase through vascular obliteration and facilitated fibrin clot formation (20). Thus, platelet concentrates such as PRF may show additional benefits on wound healing, and, because of that has been recommended for use as a bandage to cover palatal donor sites of FGG, possibly related to improvements on postoperative pain and accelerated repair of the wound. Faced with the results of the present systematic review and meta-analysis, it can be stated that the hypothesis of PRF improving healing and reducing pain was confirmed.

In the present research, the qualitative and meta-analytical (VAS = 34.84, *P* < 0.01 e CWE= 5.05, *P*<0.01) assessments corroborated with regard to both VAS and complete wound epithelialization, since they converged to reduced postoperative pain and a higher number of patients with complete wound closure in groups treated with PRF when compared with their respective controls, mainly 2 weeks postoperatively. These results are in agreement with the systematic review of Miron *et al.* 2017 (8), which concluded that the literature supports soft tissue regeneration following soft tissue regenerative procedures with PRF.

Improvements provided by PRF may be associated to different paths. More than the physical property of a plug during hemostasis, platelets are capable of stimulating proliferation and activation of cells closely involved with the repair process, such as fibroblasts, neutrophils, macrophages, and mesenchymal stem cells (21). The completion of the repair process is dependent on platelet-specific and non-specific proteins (22), growth factors such as platelet-derived growth factor (PDGF), coagulation factors, adhesion molecules, cytokines/chemokines, and angiogenic factors, all of them released and activated by platelets (21). Moreover, among the cells related to wound healing, neutrophils and macrophages also play the role of prevention of infection (23). In the early stages of inflammation, both are involved with the removal of debris and necrotic tissue, thereby preventing microbial contamination (24).

Another important component of the PRFs is the fibrin. It is a bridging molecule that supplies a tridimensional matrix in which cells related with wound closure may proliferate, organize, and play their respective roles (25). Fibroblasts and endothelial cells permeate within this fibrin network, and once they are arranged, the processes of angiogenesis and secretion of collagen begin (26).

Even faced by these extensive positive features over repair, one of the studies included in this systematic review reported no reduction of the postoperative pain in the group treated with PRF (18). VAS tends to present a wide variety of uniform results, and, therefore, although a valid method, it has limitations (27). Another topic that might be highlighted with regard to the biases in postoperative pain is the difference of prescription protocols adopted by the studies included in the present systematic review, once each medication can act directly on pain modulation.

Literature reports distinct centrifugation protocols for obtention of PRF. Kulkarni *et al.* (28) and Dohan *et al.* (22) demonstrated the same methodology for preparation of the PRF (centrifuged 10mL of blood for each tube, at 3,000 rpm for 10 minutes). Ustaoglu *et al.* (18) used as test group the protocol for obtention of T-PRF described by Tunali *et al.* (29) (centrifuged 10mL of blood for each titanium tube, at 2.800 rpm for 12 minutes). Tunali *et al.* (29) attest that the use of titanium tubes suppresses the negative effects caused by dry glass or glass-coated plastic tube. Also, titanium-activated platelet aggregation seems to present firmer network structure and longer *in vivo* resolution time than the ones formed on glass (29).

Not only modifications to the tubes are reported in the literature. Also, alterations on the rotation speed and time of centrifugation incorporated other options to the lineage of platelet concentrates. Fujioka-Kobayashi *et al.* (30) described the L-PRF (centrifuged 10mL of blood for each tube, at 2,700 rpm for 12 minutes), A-PRF (centrifuged 10mL of blood for each tube, at 1,300 rpm for 14 minutes), and A-PRF+ (centrifuged 10mL of blood for each tube, at 1,300 rpm for 8 minutes). The positive results of these protocols with regard to the release of growth factors (30) encourage the assessment of their effects on pre-clinical and clinical scenarios.

The use of any of the blood derivate depends on the compliance of the patient, so, individuals who are afraid of needles preclude this procedure. Even with the limitations assigned to platelet concentrates, some non-biological advantages shall be emphasized about PRF. The protocol for preparation of this specific product may be considered of low-cost and easy to perform. Furthermore, PRF doesn’t require biochemical manipulation of the blood samples.

Despite the consistency and strength of the qualitative outcomes, the absence of standardized control group among studies could represent a limitation of the quantitative analysis while comparing results. Hence, the positive results obtained with PRF presented by this meta-analysis shall be interpreted embracing this situation. Further randomized clinical trials adopting standardized control groups of palatal wound healing might be carried in order to provide substantial data for confirmation of the effectiveness of PRF, and to increase the number and reliability of assessments evaluating this treatment.

Within the limits of the present research, the qualitative synthesis of six studies combined with the meta-analysis of two studies evaluating pain and two studies evaluating complete wound epithelialization infers that the use of PRF reduces the postoperative pain and induces earlier epithelialization of the palatal donor site of FGG.
